# The microbial aerial thread: co-restoring source and sink across dryland-urban gradients

**DOI:** 10.3389/frmbi.2026.1965062

**Published:** 2026-10-19

**Authors:** Albert Barberán, Elise S. Gornish

**Affiliations:** 1 Department of Environmental Science, University of Arizona, Tucson, AZ, United States; 2 School of Natural Resources and the Environment, University of Arizona, Tucson, AZ, United States

**Keywords:** aeromicrobiology, bioaerosols, drylands, dust dispersal, ecological restoration, urban ecology

## Abstract

Dryland ecosystems serve as major sources of atmospheric bioaerosols through aeolian transport of dust-associated microorganisms. These microbial communities are dispersed across vast distances, connecting dryland soils and vegetation to distant recipient ecosystems, particularly urban areas. This review synthesizes current understanding of microbial ecology in arid soils, microbial dispersal through dust, and interactions with urban ecosystems. We propose that restoration efforts should move beyond isolated, site-level interventions toward integrated strategies that account for the atmospheric connectivity between dryland source areas and urban sink environments. Practical restoration approaches, including dryland soil stabilization to reduce harmful dust emissions, microbial inoculant development, and urban green infrastructure designed to function as dispersal corridors, can generate cross-boundary synergies that enhance ecological resilience across the dryland–urban gradient. By reconceptualizing cities as nodes within broader ecological networks linked by microbial dispersal, and by leveraging insights from dryland ecology to guide urban restoration under resource-limited conditions, this framework offers a pathway for co-restoring connected landscapes in an era of accelerating urbanization and desertification.

## Introduction

Dryland microbial communities extend far beyond the soil and their plant hosts. Through aeolian transport, bacteria, fungi, and viruses are lofted into the atmosphere and dispersed across vast distances (Kellogg and Griffin, 2006). These bioaerosols do not merely drift; during atmospheric transport, these assemblages undergo strong environmental filtering, and upon deposition they facilitate global genetic exchange and drive the ecological dynamics of downwind ecosystems (Joly et al., 2013; Amato et al., 2023). Crucial among these receiving environments are urban areas, which host more than half of the global population and present a contrasting, yet intimately connected, ecological context. Urban areas are characterized by unique soil formations (Pavao-Zuckerman, 2008), which require flexible technologies to successfully conserve and restore these systems in the face of environmental change (Klaus and Kiehl, 2021). Recognizing cities as dynamic socio-ecological systems emphasizes the necessity for intentional urban design and management that considers not only microbial inputs through atmospheric deposition, but also the local environmental conditions that determine whether deposited microorganisms survive, establish, and contribute to microbial diversity, ecosystem functioning, or human health (Catalano et al., 2021).

This review synthesizes current understanding of microbial ecology in arid soils, their dispersal through dust, and their interactions with urban ecosystems. By considering atmospheric dispersal, connectivity, and cross-ecosystem interactions, management (specifically, ecological restoration or functional rehabilitation) and urban planning can move beyond isolated, local-scale interventions toward strategies that enhance and mutually reinforce ecological resilience and service provision in dryland and urban ecosystems. Through this lens, cities become nodes in a broader ecological network rather than endpoints of degradation, and microorganisms are not merely passive components of the environment but active agents linking soils, air, vegetation, and human health across scales.

## The waste land: microorganisms in arid soils

Drylands cover approximately 40% of the terrestrial surface and support more than 38% of the global human population. These proportions are expected to increase as a result of ongoing desertification and population growth (Maestre et al., 2016). Because vegetation cover is typically sparse in these regions, soil and its microbial communities play a critical role in regulating key ecosystem functions such as soil erosion, water infiltration and retention, soil aggregation and stability, and plant nutrient availability (Pointing and Belnap, 2012). However, soil degradation increasingly threatens these functions by reducing biodiversity, plant productivity, and soil carbon storage, while increasing dust-related health risks such as allergies and traffic accidents from dust storms (Bestelmeyer et al., 2015; Hoover et al., 2020). In drylands, limited water availability, low soil organic matter, and constrained plant growth can prolong ecosystem recovery following disturbance (Steven et al., 2021), and, in some cases, trigger irreversible regime shifts (Berdugo et al., 2020).

Although soil erosion and dust production are commonly used indicators of land degradation, arid soils are naturally exposed to the action of the wind due to sparse vegetation (Field et al., 2010; Duniway et al., 2019). Furthermore, in many arid regions, particularly coastal deserts such as the Atacama, fog mediates transport and wet deposition while providing moisture that may enhance post-deposition survival (Fernández-Martínez et al., 2019). Overall, microbial airborne dispersal via dust represents an important ecological process in arid and semi-arid ecosystems that warrants careful consideration (Kellogg and Griffin, 2006).

## Ask the dust: aeromicrobiology and microbial dispersal

Dust consists of fine particulate material that is removed from the terrestrial surface by wind erosion and is small enough to be suspended in the atmosphere (Field et al., 2010). Microorganisms can be lifted by the wind and dispersed through air (Kellogg and Griffin, 2006) and thus, are ubiquitous and diverse in dust, where they can represent a substantial fraction of aerosolized particles (Després et al., 2012).

Although the atmosphere typically contains low microbial cell densities (Burrows et al., 2009; Amato et al., 2023), dust particles are often enriched in stress-tolerant bacterial taxa (Aalismail et al., 2019; Archer et al., 2023). These airborne microorganisms persist under intense UV radiation, desiccation, oxidative stress, and temperature fluctuations through adaptations including spore formation, GC-rich genomes, efficient DNA repair, photoprotective pigments, cold-shock proteins, endolithic lifestyles, and protective exopolysaccharides (Tripp et al., 2016; Choudoir et al., 2018; Šantl-Temkiv et al., 2022). Fungi, in contrast, often disperse mainly as free-floating spores rather than attached to soil particles (Schiro et al., 2022). In addition to soils, the phyllosphere is a major source of airborne microbes, explaining the frequent detection of plant-associated bacteria (Favet et al., 2013), and fungal pathogens in dust (Dietzel et al., 2019), with potential consequences for agriculture and food security. Furthermore, vegetation cover can influence soil stability, soil moisture, and soil erosion, therefore shaping the amount of microbial biomass aerosolized and its composition (**Figure 1**).

**Figure 1:**
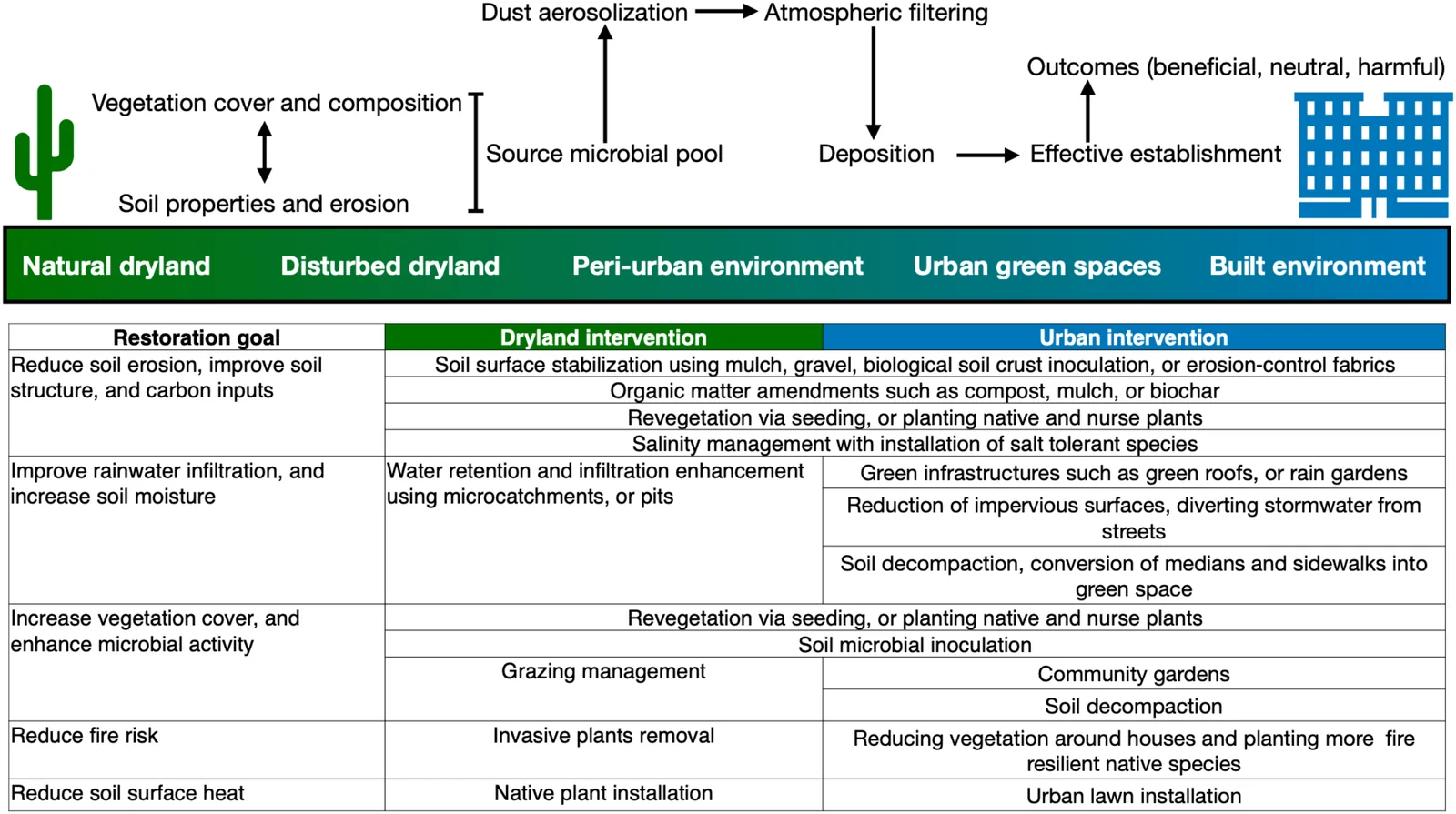
Conceptual figure integrating the gradient from natural drylands to build environments, the main ecosystem properties and ecological processes participating in their connectivity, and an overview of soil restoration interventions in dryland and urban ecosystems organized by shared restoration goals.

Dust storms (Vila‐Costa et al., 2013) and wildfires (Kobziar et al., 2022) can transport microorganisms across thousands of kilometers away from their sources, affecting distant recipient ecosystems, including urban environments (Kellogg and Griffin, 2006; Barberán et al., 2014; Cáliz et al., 2022). For example, Saharan dust is transported approximately 2,600 km across the Atlantic Ocean to eastern South America, with substantial deposition occurring over the Amazon Basin (Kellogg and Griffin, 2006). The subsequent fate of these microorganisms may range from rapid mortality or dormancy to transient activity and, under suitable conditions, ecological establishment (Aalismail et al., 2019; Archer et al., 2023). This distinction is critical because the detection of microbial DNA in airborne or deposited dust demonstrates transport but does not, by itself, establish cell viability, metabolic activity, population growth, or functional effects in the receiving ecosystem (Kellogg and Griffin, 2006). The intersection of dust-associated bioaerosols and urban environments is particularly relevant given that a substantial proportion of the world’s cities are located in dryland regions, and this proportion is projected to increase (Grimm et al., 2008). Research comparing bioaerosol communities between dust source regions (such as the Gobi Desert) and urban deposition areas has demonstrated that dust events significantly increase the concentration and diversity of airborne microorganisms in cities, and can alter the microbial community composition of urban atmospheres (Dong et al., 2022). These findings highlight how rapidly expanding dryland cities face a dual challenge: increasing exposure to dust-borne microbiota as desertification intensifies, while simultaneously experiencing the ecological consequences of urbanization on local microbial communities.

## Invisible cities: microbial communities in urban ecosystems

Urban ecosystems are generally characterized by high population density and extensive built infrastructure that cover much of the surface and subsurface environment. Various urban disturbances, such as air, water and soil contamination, gas emissions and habitat fragmentation can impact the climate, environmental health, and the biotic communities within, around and beyond urban boundaries (Bai et al., 2017). One prominent ecological consequence of these transformations is biotic homogenization, a process whereby biological communities become increasingly similar due to landscape simplification, species invasions, and other anthropogenic pressures (McKinney, 2006). This reduction in geographic variability has been documented in microbial communities, indicating that urbanization can homogenize not only plants and animals but also the invisible microbial world (Barberán et al., 2015a; Epp Schmidt et al., 2017; Delgado-Baquerizo et al., 2021). Clearly, restoration or functional rehabilitation (i.e., recovering selected ecological functions without necessarily restoring the ecosystem’s original species composition) approaches that attempt to address chronic degradation in urban systems should target the maintenance and reinvigoration of diverse microbial communities (Farrell et al., 2020).

Addressing these challenges requires a shift from viewing cities solely as sites of ecological impact to recognizing them as dynamic socioecological systems that can be intentionally designed and managed to promote environmental and public health (Catalano et al., 2021). In this context, the built environment microbiome represents a critical yet often overlooked component of urban ecosystems (Touceda-Suárez et al., 2025) and their management (Mills et al., 2017). Because urbanization reshapes biodiversity patterns, biogeochemical cycles, and ecosystem services (Bai et al., 2017), incorporating microbial perspectives into urban design is essential for maintaining functional and healthy environments. Achieving this goal demands multidisciplinary collaboration among urban planners, architects, public health experts, microbial ecologists, and policymakers to ensure that built environments foster beneficial microbial exposures while minimizing health risks. Such integrative approaches must also consider the strong ecological linkages between cities and their surrounding landscapes (recognizing that urban and peri-urban systems are themselves located within drylands), including the movement of organisms and materials across urban–dryland gradients (**Figure 1**). These considerations naturally lead to the need for restoring and managing connected habitats to enhance resilience, and ecosystem service provision in an increasingly urbanized world (Watkins et al., 2020).

## There and back again: co-restoring source and sink

Efforts to restore degraded landscapes and redesign urban ecosystems have traditionally focused on single sites or local scales (Gilby et al., 2021), often implicitly treating communities as closed and ecologically isolated units. This local focus is not solely conceptual; it also reflects practical constraints on funding, labor, and institutional capacity that affect urban restoration projects (Busbridge et al., 2021) and other systems (Li and Gornish, 2020). Although this site-level approach has yielded valuable insights and tepid success (Sasaki et al., 2018), it overlooks the fact that most ecological communities are embedded within broader spatial networks and are continuously shaped by dispersal processes (Swan and Brown, 2017). Recognizing and integrating the linkages between drylands and urban areas into urban restoration strategies is essential for accommodating the ongoing exchange of microbial communities between these ecosystems.

Here, we focus on dryland ecosystems as critical source habitats, given their spatial extension, their capacity to mobilize microorganisms via wind and dust, and their close geographic proximity to rapidly expanding urban populations, although it generally should be considered a gradient involving natural drylands, degraded/disturbed drylands, peri-urban environments, urban green spaces, and built environments (**Figure 1**). Dust emissions from drylands can negatively affect human health and environmental quality in urban ecosystems. For example, the soil-dwelling fungi *Coccidioides* can become airborne and cause coccidioidomycosis (Valley fever) upon inhalation (Sprigg et al., 2014), while unvegetated mine tailings in arid regions may disperse metal-contaminated particles that contribute to environmental toxicity and potentially promote antimicrobial resistance in nearby high population density areas (Baker-Austin et al., 2006). At the same time, exposure to diverse environmental microbiota can also provide important health benefits. In children, contact with less urbanized environments has been associated with enhanced immune regulation, reduced risk of allergies and inflammatory disorders (Rook, 2013; Stein et al., 2016), and distinct gut microbiomes (Sbihi et al., 2019) compared to those raised in highly urban settings.

Ultimately, the composition and dynamics of microbial communities in urban and surrounding ecosystems are not merely the outcome of natural processes (Barberán et al., 2015b; Chen et al., 2021); they are also shaped by human decisions in designing and managing the built environment (Robinson et al., 2021; Touceda-Suárez et al., 2025). Choices regarding vegetation cover, green space connectivity, building materials, and water management influence which microorganisms thrive, how they disperse, and the extent to which humans are exposed to beneficial or harmful taxa (Kembel et al., 2012; Laforest-Lapointe et al., 2017; Rai et al., 2021). For instance, human-associated potential pathogens and antimicrobial resistance genes are generally more abundant in urban soils, freshwater systems, and indoor environments than in natural ecosystems (Ramirez et al., 2014; Charlop-Powers et al., 2016; Numberger et al., 2022). Indoors, microbial communities are further shaped by factors such as ventilation, airflow, and human occupancy (Barberán et al., 2015a; Rai et al., 2021), with poorly ventilated spaces often harboring higher abundances of human-associated pathogens (Kembel et al., 2012). Besides microbial exposure, soil nutrients, as well as organic matter, are often bound to finer soil particles, meaning that dust transport can deplete fertility in source areas while simultaneously enriching sink environments (Li et al., 2007).

A recent study in Urumqi (China) found that ecological restoration and targeted land-use policies strengthened carbon sink capacity in restored suburban areas while industrialized zones remained carbon sources, highlighting how material and energy dynamics are redistributed across the urban–dryland continuum (Zhang et al., 2026).

As a consequence, restoration interventions should be evaluated not only locally but also for their cross-boundary ecological and health effects, and their potential interactions and synergies (**Figure 1**). For instance, actions that reduce dust emissions in drylands also benefit urban respiratory health (although dust microbiota can also lead to neutral or harmful outcomes), while urban soil restoration improves stormwater infiltration, heat mitigation, and biodiversity, which in turn reduces pressure on surrounding dryland resources (Duniway et al., 2019). Furthermore, while microbial inocula from restored drylands (particularly, those with stress-tolerant traits) can disperse atmospherically into cities, potentially enriching urban soils with beneficial taxa adapted to aridity and nutrient cycling (Muñoz-Rojas et al., 2018), urban green infrastructures and peri-urban restoration zones can function as microbial dispersal corridors, connecting dryland ecosystems regardless of administrative boundaries (Joyner et al., 2019). However, not all interactions are synergistic, and not all properties or processes can be directly manipulated (e.g. wind speed or direction). Trade-offs between provisioning services (food production) and regulating services (carbon storage, water purification) exist (Zhao et al., 2022). The key is to manage the dryland-urban gradient as an integrated system, leveraging atmospheric microbial dispersal, maintaining structural connectivity, and balancing the inherent trade-offs between ecosystem services through spatially explicit, ecologically informed planning.

Finally, cities can function as testbeds for restoration innovations such as drought-adapted soil amendments, microbial inoculants, or low-water vegetation strategies that later can scale into surrounding drylands. Conversely, dryland ecosystems can act as models for guiding urban restoration. The microbial communities and plant–soil interactions that naturally develop in these arid environments offer reference points for achieving resilient ecosystem function under resource-limited conditions. This helps shift urban restoration goals away from maximizing productivity or greenness toward maintaining stability, multifunctionality, and resource efficiency under chronic water limitation. In this way, integrating sustainable urban design into restoration planning becomes an active tool not only for shaping the physical environment, and enhancing ecological resilience across urban–dryland gradients, but also for guiding the invisible, yet ecologically significant, microbial communities that connect cities to their broader landscapes.
